# A case report of melioidosis complicated by infective sacroiliitis in Sri Lanka

**DOI:** 10.1186/s40794-018-0073-5

**Published:** 2018-09-19

**Authors:** A. K. T. M. Karunarathna, S. A. Mendis, W. P. D. P. Perera, Geethika Patabendige, A. S. Pallewatte, Aruna Kulatunga

**Affiliations:** National Hospital, Colombo, Sri Lanka

**Keywords:** Melioidosis, *Burkholderia pseudomallei*, Musculoskeletal manifestations of melioidosis, Multiple intramuscular abscesses, Sacroiliitis, Intensive phase, Eradication phase, SIADH

## Abstract

**Background:**

Melioidosis is an infection caused by a facultative intracellular Gram-negative bacterium, *Burkholderia pseudomallei*. It can present as septicemia, localized infection with/without septicemia, asymptomatic infections, ulcers, pneumonia, visceral abscesses, neurological infection, musculoskeletal infections and can involve any organ.

**Case presentation:**

A 56 year old Sri Lankan diabetic female presented with fever, chills and rigors for 2 weeks. She also had malaise and loss of appetite, but no other features. On examination, she was febrile (temperature was 101.4 ^0^ F) and rest of the examination was unremarkable. Her blood culture was positive for *Burkholderia pseudomallei* and she was started on IV antibiotics, on day 3. During her 2nd week of hospital stay, she developed right sided low back pain with buttock pain, right hip joint pain and restricted hip joint movements suggestive of right sacroiliitis. CE CT and MRI scans confirmed the diagnosis of right iliopsoas abscesses and right sacroiliitis.

Incision and drainage was performed and a pigtail catheter was left in place for continuous drainage of abscesses. Her intensive phase was initiated with IV ceftazidime 2 g every 6 h for 12 days, then changed over to IV meropenem 2 g every 8 h together with oral co-trimoxazole. 2 weeks later, oral co-trimoxazole was replaced by oral doxycycline for another 6 weeks (due to transient pancytopaenia). She made a complete and uneventful recovery with oral co-trimoxazole for another 6 months, in her eradication phase.

We report this case to show the importance in early diagnosis of melioidosis, and to consider it in the differential diagnosis of multiple abscesses and to emphasize the importance in suspecting melioidosis as a causative agent in infective sacroiliitis.

**Discussion:**

Melioidosis can have 2 major presentations; acute infection (symptoms lasting less than 2 months) and chronic infection (symptoms lasting more than 2 months). Musculoskeletal melioidosis is a well-recognized manifestation of the disease, which can manifest as soft tissue abscesses, septic arthritis, spondylitis, sacroiliitis and osteomyelitis.

Management of melioidosis consists of 2 phases. The intensive phase and the eradication phase. These are aimed at the importance of rapidly treating the septicemia, the need of eradication of the persistent disease and the prevention of recurrent infections or relapses. The intensive phase consists of minimum 10–14 days of IV antibiotics: IV ceftazidime or IV carbapenem (meropenem/ imipenem). Eradication phase should be followed by 3–6 months of oral co-trimoxazole alone or in combination with oral doxycycline/ oral amoxiciliin-clavulanic acid.

## Background

Melioidosis is an infection caused by a facultative intracellular Gram-negative bacterium, *Burkholderia pseudomallei*, which is a saprophytic soil bacterium. It is most predominant in South-East Asia, South Asia, Nothern Australia & China.

We report a case of a 56 year old female diabetic patient who presented with fever for 2 weeks, associated with multiple intramuscular abscesses and right sided sacroiliitis, caused by melioidosis. We report this case to show the importance in early diagnosis of melioidosis and consideration of melioidosis in the differential diagnosis of patients presenting with multiple abscesses or an atypical presentation of infective sacroiliitis.

## Case presentation

A 56 year old Sri Lankan female presented with fever, chills and rigors for 2 weeks. She was diabetic and her glycaemic control was fairly good with oral hypoglycaemic drugs: fasting blood sugar was 112 mg/dl and HbA1C was 5.7%. At presentation, fever was associated with constitutional symptoms like malaise and loss of appetite, but she didn’t have any other symptoms on systemic review. She is a housewife, not engaged in gardening or farming.

On admission, upon examination, she was febrile (temperature was 101.4 ^0^ F), did not have lymphadenopathy, oral ulcers, uveitis, peripheral signs of infective endocarditis, rashes or hepatosplenomegaly. Rest of her clinical examination was unremarkable.

**Fig. 1:**
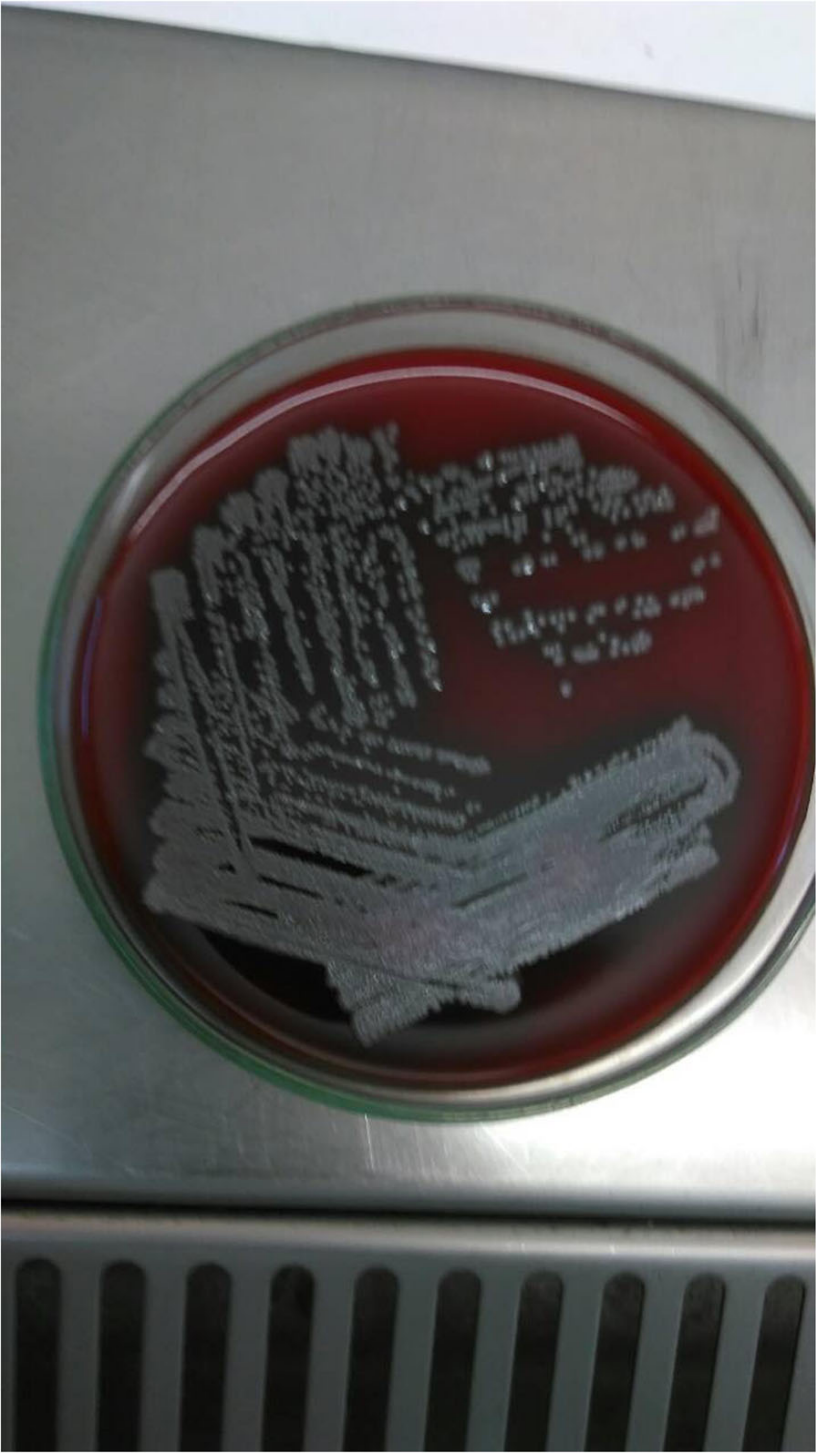
Blood agar: Smooth, tiny colonies initially which become chalky white after few days

**Fig. 2:**
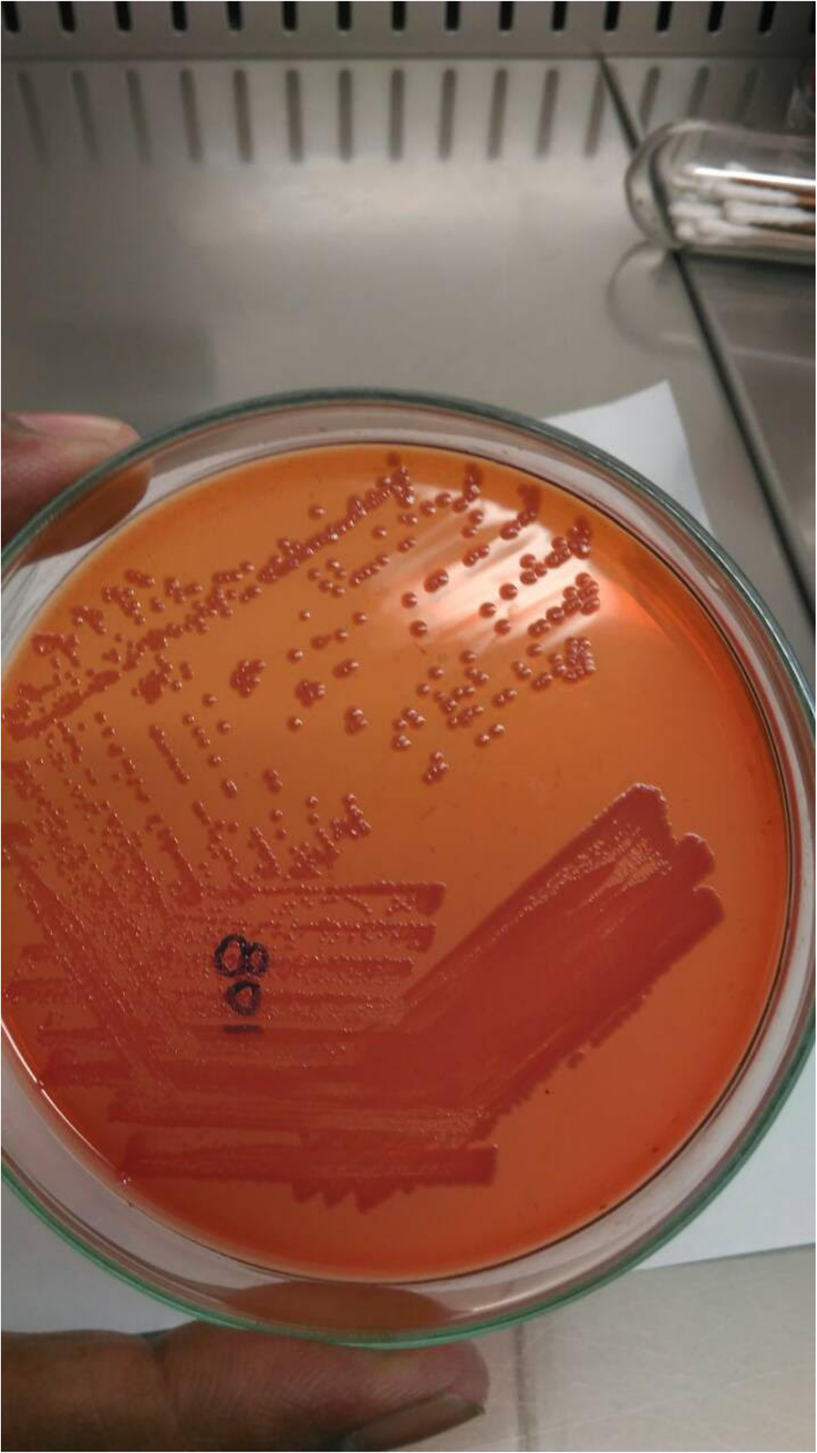
MacConkey Agar: Pink or colourless colonies

On the third day of admission, blood culture reported positive with the growth of *Burkholderia cepacia*. She was started on IV ceftazidime 2 g every 6 h, with the suspicion of *Burkholderia pseudomallei* and melioidosis serology was ordered. Colony morphology consisted of grey-white small, smooth colonies. Gram stain showed gram negative slender rods with bipolar staining, antibiotic susceptibility pattern gave clues to the microbiological diagnosis of *Burkholderia pseudomallei*, sensitive for ceftazidime and meropenem and resistant to gentamycin (Figs. 1 and 2). The melioidosis antibody titer Ig M was 1:640, measured by enzyme linked immunosorbent assay (ELISA). The isolate was confirmed as *Burkholderia pseudomallei* by PCR the following day.

**Fig. 3:**
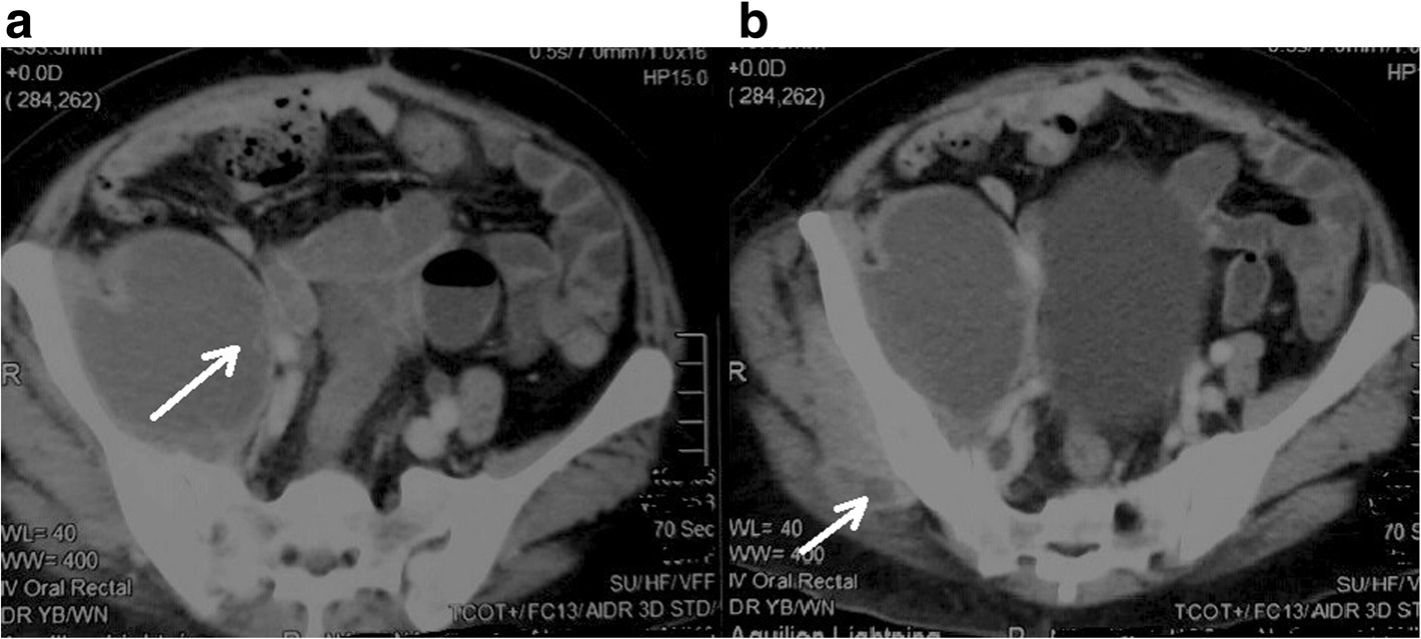
**a** and **b** Axial view of Contrast Enhanced CT Abdomen and pelvis images showing cystic lesion with an enhancing wall suggesting abscesses in right psoas, iliacus muscles extending to gluteus muscles(white arrows)

She was continued on IV ceftazidime 2 g every six hours. During her 2nd week of hospital stay (24th day of fever), she developed right hip joint pain, right sided low backache, right buttock pain, difficulty walking and restricted right hip joint movements. She did not have any other joint involvement or history of arthritis. She did not have past history of tuberculosis or significant contact history of tuberculosis. She did not have change of bowel habits, red eyes to suggest inflammatory bowel disease. She didn’t have any skin rashes to suggest psoriasis or vasculitis or recurrent oral ulcerations to suggest behcet’s disease. She did not have a history of raw milk ingestion to suggest brucellosis. She didn’t have family history of arthritis or autoimmune diseases. By that time, her right hip joint movements were restricted due to pain, she had severe tenderness over right sacroiliac joint and sacroiliac stretch maneuvers were positive for a clinical diagnosis of right sacroiliitis. She had a neutrophilic leukocytosis, with high inflammatory markers. Ultrasound Scan of the hip joint detected a large fluid collection measuring 5 cm × 18 cm involving the right iliopsoas, without extension into hip joint. A Contrast Enhanced CT scan (CE CT) of Chest, abdomen and pelvis showed multiple intramuscular abscesses over right psoas, iliacus, gluteus and obturator internus (the largest abscess measured 18 cm × 4 cm over right psoas and iliacus (Fig. 3)). There were no visceral abscesses in the liver or spleen. Initially US guided aspiration and drainage was carried out to drain the iliopsoas abscesses (by the 28th day of fever), followed by a pig tail catheter drainage over 5 days, since it was a large collection. The aspirated fluid culture was also positive for *Burkholderia pseudomallei*.

**Fig. 4:**
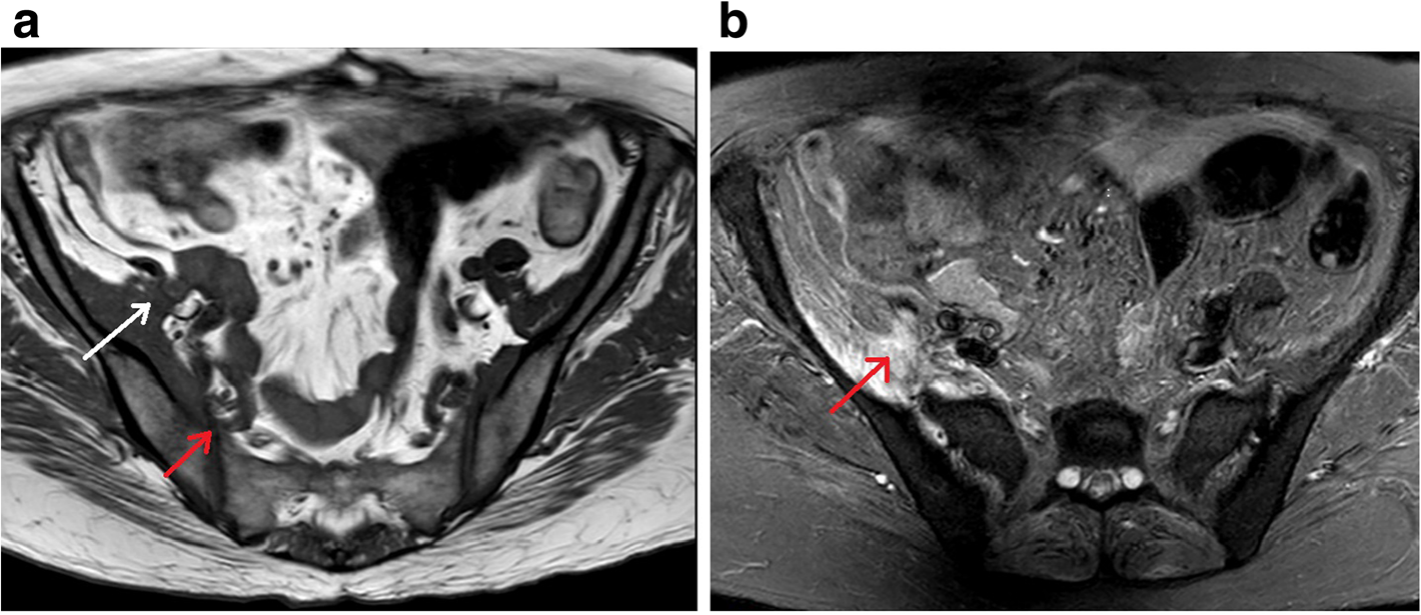
Coronal view of MRI sacroiliac joints: (during intensive phase treatment) **a** T1 Weighted Image showing thickening of right internal iliacus muscle(white arrow) and erosion of anterior joint space of right iliac joint(red arrow), **b** Fat supressed (STIR) image showing thickening of right internal iliacus muscle, oedema of fat planes anterior to right iliac bone and sacrum with extension into anterior joint space of right iliac joint(red arrow)

One week post aspiration, she had an MRI scan of spine and sacroiliac joints: which showed resolving infection in right iliacus muscle, with a thin collection of fluid and muscle oedema. It also showed right sacroiliitis with surrounding marrow oedema and mild reactive bone changes (Fig. 4). We didn’t aspirate fluid from sacroiliac joint, since an alternative cause for sacroiliitis was unlikely, melioidosis was the likely cause for her right sacroiliitis.

Fever, multiple abscesses with sacroiliitis, pointed towards the musculoskeletal involvement of melioidosis. Her sacroiliitis was infectious in nature, so inflammatory causes like psoriatic arthritis, ankylosing spondylitis and inflammatory bowel disease were unlikely. Anyway we wanted to exclude co-infection of melioidosis with tuberculosis/ brucellosis/ fungal infections and immunosuppressed states like AIDS (Acquired Immuno Deficiency Syndrome).

Her Mantoux test was negative, chest X ray was normal, sputum for acid fast bacilli were negative and pus drained from abscesses didn’t yield any growth on bacterial cultures, AFB cultures or fungal cultures. Brucella and HIV antibodies were also negative.

Despite treatment with IV ceftazidime, her fever spikes continued attributable to the complications with intramuscular abscesses and sacroiliitis. Her Melioidosis antibody titers obtained 2 weeks apart increased from 1/640 to 1/10240. Thus IV ceftazidime (given for 12 days) was changed over to IV meropenem 2 g every 8 h with oral co-trimoxazole 3 tablets (each tablet = 400 mg of sulfamethoxale and 80 mg of trimethoprim) every 12 h was added on the 28th day of fever.

Results of some of the relevant investigations are shown below, given in a Table 1.

**Table 1:** Important results of the investigations done

Investigation	Before starting treatment (day 1 of admission)	During treatment in intensive phase		After treatment of 6 months of intensive phase	Normal range
Haemoglobin (g/dL)	8.8	8.5	7.9	11.6	11–13
White cell count(10^9^/L)	22.3	19.6	2.8		4–11
Neutrophil percentage	81%	81%	62%		
Platelet count (10^9^ /L)	547	452	113		
CRP(mg/L)	143	108	58	< 6	0–6
ESR(mm/1st hour)	125	65	50	27	
Serum creatinine (μmol/L)	133	111			60–120
Serum sodium (mmol/L)	138	130	126	136	135–148
Serum potassium (mmol/L)	4.5	4.4	4.1	3.6	3.5–5.1
AST (U/L)	38	40	34	25	< 40
ALT (U/L)	27	39	17	20	< 40
ALP (U/L)	116	90		116	30–120
Serum total proteins(g/L)	69		59	68	61–80
Serum Albumin(g/L)	27		28	28	36–50
Serum Globulin(g/L)	42		31	40	22–40

According to the recent international guidelines, in deep seated abscesses, duration of intensive phase of melioidosis is extended and retimed from the date of most recent drainage of *Burkholderia pseudomallei.* [1–3]. Therefore our patient’s IV therapy was extended additional 14 days of abscess drainage. She developed pancytopenia, during the second week of treatment with co-trimoxazole. Her haemoglobin was 7.9 g/dL, white cell count was 2.8 × 10^9^/L and platelet count was 113× 10^9^ /L. This was changed into oral doxycycline 100 m grams every 12 h to avoid bone marrow suppression. Her intensive phase was continued with IV meropenem 2 g every 8 h another 6 weeks together with oral doxycycline. During the 6th week of intensive phase treatment, she also developed Syndrome of Inappropriate Secretion of ADH (SIADH), with persistent hyponatraemia (120-135 mmol/L), urinary sodium of 92 mmol/L, and serum osmolality 264mosm/kg and urine osmolality of 389mosm/l. She responded within 1 week with fluid restriction therapy alone, without change of medical management.

**Fig. 5:**
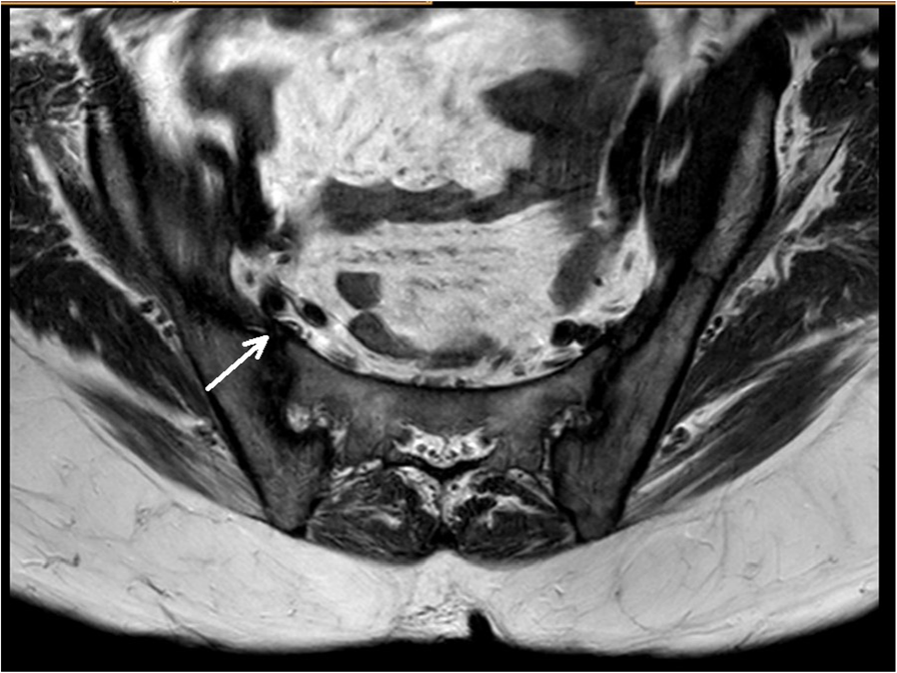
Coronal view of MRI sacroiliac joints: (following completion of intensive phase treatment) There is reduction of oedema over right internal iliacus muscle, iliac bone and sacrum with normal right sacroiliac joint(white arrow)

She was restarted on oral co-trimoxazole 4 tablets every 12 h, at the end of intensive phase treatment, (each tablet = 400 mg of sulfamethoxazole and 80 mg of trimethoprim) and discharged her on eradication phase (since cotrimoxazole is the best drug in eradication phase, it was restarted and she didn’t develop pancytopaenia this time). She was followed up monthly for 6 months and she made an uneventful recovery at the end of eradication phase. Follow up MRI Scan of sacroiliac joints done after 2 months showed marked reduction of infection, oedema and sacroiliitis and by that time, she was completely asymptomatic (Fig. 5).

## Discussion

Melioidosis is caused by *Burkholderia pseudomallei*, which is a facultative intracellular Gram-negative, saprophytic bacterium, commonly found in soil or contaminated water [4–7]. It has previously been called as *Pseudomonas pseudomallei*. It is most predominant in South-East Asia, South Asia, Nothern Australia & China [5, 6, 8–10]. Sri Lanka lies in the melioidosis-endemic belt between 5°N and 10°N surrounded by countries known to be endemic to melioidosis [4].

The populations at risk are those who have occupational exposure to wet soil or surface water in the form of farming, agriculture, gardening, fishing, manual labour, land surveying, building construction and immunocompromised states such as diabetes, alcoholism, chronic renal failure, chronic lung disease and HIV/AIDS [3, 4, 7, 8, 10–12].

Direct inoculation(specially through breaks in skin) is known to be the major mode of transmission of infection. Inhalation, ingestion, person to person transmission are another common modes of transmission and sexual transmission, vertical transmission at child birth have also been reported [6, 7, 11–14]. It has a significant mortality rate despite treatment and also it’s known to cause reinfection and recurrences [3, 7, 14, 15].

Melioidosis can have 2 major presentations; acute infection (symptoms lasting less than 2 months) and chronic infections (symptoms lasting more than 2 months) [8, 12, 16]. Melioidosis can present as septicemia, localized infection with/without septicemia, asymptomatic infections, ulcers, pneumonia,visceral abscesses, neurologic infection, musculoskeletal infections [6, 8, 9].

Musculoskeletal melioidosis is a well-recognized manifestation of the disease [7, 12]. It can manifest as soft tissue abscesses, septic arthritis, spondylitis, sacroiliitis and osteomyelitis [7, 12]. Our patient had bacteremia with multiple musculoskeletal areas of involvement: iliopsoas abscesses and right sacroiliitis.

There should be a high degree of suspicion of melioidosis, when patients present with multiple abscesses in the appropriate geographic area with potential exposure history [6, 10]. Deep abscesses should be managed with USS/CT scan guided incision and drainage when possible. If complete drainage is not achieved, pigtail catheter drainage could be applied. Isolation of the organism can be achieved with specimens of blood, pus, wound swabs, urine and sputum. It is important to note that the manual identification kits and automated microbial identification systems generally do not identify this organism to the species level as *Burkholderia pseodomallei*. When we have high degree of suspicion of *Burkholderia pseudomallei,* we should specifically look for the colony morphology, Gram stain appearance, antibiotic susceptibility pattern of the microbe, melioidosis antibody level and PCR. Even though there was no delay in initiation of treatment in our patient, close resemblance of the organism to Pseudomonas is a leading cause of delayed identification and delayed initiation of treatment worldwide [5]. Underdiagnosis or misdiagnosis have been reported in many instances due to lack of familiarity with this organism, lack of knowledge of similar characteristics of the organism with other Gram negative bacteria, especially of the Pseudomonas group and lack of microbiological experiences [6, 11, 12]. Serological investigations (haemagglutination /ELISA) may have a place in diagnosis of melioidosis, especially when cultures are negative [5, 7]. There have been presumptive treatment with antituberculosis drugs specially in tuberculosis endemic areas for patients with arthritis, lymphadenitis, spondylitis, pneumonia and visceral abscesses later diagnosed as melioidosis [11, 12].

When patients present with backache, buttock pain, associated with fever, with clinically evident sacroiliitis, one must have a high degree of suspicion towards an infective cause of sacroiliitis. Tuberculosis and brucellosis are among the main infectious causes of sacroiliitis [11, 17]. To the best of our knowledge, sacroiliitis due to melioidosis is rarely reported worldwide.

Management of melioidosis consists of 2 phases -the intensive phase and the eradication phase. These are aimed at the importance of rapidly treating the septicemia, the need for eradication of the disease and prevention of relapses [12, 15, 18]. The combination of agents used, duration of therapy and need for adjunct modalities depends on the type, severity and antimicrobial susceptibility of the infection [18]. The intensive phase consists of 10 days–14 days (which can be extended even for 4 weeks, if clinically indicated) of IV antibiotics: IV ceftazidime or IV carbapenem (meropenem/ imipenem). If there are fluid collections (including skin abscess/septic arthritis), bone or central nervous system involvement, co-trimoxazole, doxycycline or amoxicillin-clavulanate should be added early during the intensive phase for tissue penetration [3]. Subsequently the eradication phase should be with another 3–6 months of oral co-trimoxazole (Trimethoprim-sulfamethoxazole) alone or in combination with oral doxycycline/ oral amoxyciliin-clavulunate [3, 15, 18]. Melioidosis causes significant case fatalities, recurrences and reinfections [18]. Many patients with melioidosis are reported to have had hyponatraemia (as in our patient), with /without SIADH. Whether it’s due to the drugs or the disease itself is to be studied further.

We report this case since our patient had musculoskeletal manifestations of melioidosis with iliopsoas abscesses, right sacroiliitis with bacteremia and made a complete uneventful recovery, with timely diagnosis and proper management. We wish to emphasize the importance of a high degree of suspicion in making the diagnosis of melioidosis as well as the need for adequate microbiological knowledge and competence in identifying the organism correctly.

## Conclusions

Melioidosis is an emerging infection, mostly in tropical countries, with varied clinical manifestations. Musculoskeletal involvement of melioidosis is a known presentation. To the best of our knowledge, melioidosis complicated with sacroiliitis is rarely reported. Management of melioidosis is comprised of intensive and eradication phases. Patients can make a complete recovery (as in our patient), with proper treatment and follow up.

## Abbreviations

AFB: Acid Fast Bacilli
ALT: Alanine Transaminase
AST: Aspartate Transaminase
CE CT: Contrast Enhanced Computer Tomography
co-trimoxazole: trimethoprim/sulfamethoxazole
CRP: C-Reactive Protein
ELISA: enzyme linked immunosorbent assay
ESR: Erythrocyte Sedimentation Rate
MRI: Magnetic Resonance Imaging
PCR: Polymerase Chain Reaction
SIADH: Syndrome of Inappropriate Secretion of Antidiuretic Hormone
